# CLINICAL RELEVANCE OF ESOPHAGEAL MOTILITY DISORDERS AFTER BARIATRIC SURGERY: A PROSPECTIVE STUDY BASED ON HIGH-RESOLUTION IMPEDANCE MANOMETRY

**DOI:** 10.1590/0102-6720202400048e1842

**Published:** 2024-12-02

**Authors:** Lucas dos Santos DIFANTE, Eduardo Neubarth TRINDADE, Antonio de Barros LOPES, Eduardo Ferreira MARTINS, Isadora Bosini REMUS, Manoel Roberto Maciel TRINDADE

**Affiliations:** 1 Universidade Federal do Rio Grande do Sul, Hospital de Clínicas de Porto Alegre, Department of Digestive Surgery – Porto Alegre (RS), Brazil; 2 Universidade Federal do Rio Grande do Sul, Hospital de Clínicas de Porto Alegre, Department of Gastroenterology – Porto Alegre (RS), Brazil.

**Keywords:** Obesity, Bariatric Surgery, Manometry, Esophageal Motility Disorders, Obesidade, Cirurgia Bariátrica, Manometria, Transtornos da Motilidade Esofágica

## Abstract

**BACKGROUND:**

There is recent evidence showing that obesity is associated with gastroesophageal reflux disease and esophageal dysmotility, although symptoms are not always present.

**AIMS:**

This is a prospective study based on high-resolution manometry findings in bariatric surgery candidates and their correlation with postoperative dysphagia.

**METHODS:**

Manometric evaluation was performed on candidates for bariatric surgery from 2022 to 2024. The examination was conducted according to the protocol of the fourth version of the Chicago Classification, including different positions and provocative maneuvers to confirm the diagnosis of dysmotility. Patients were followed for 90 days after surgery to verify the occurrence of dysphagia or difficulty adapting to the diet.

**RESULTS:**

High-resolution manometry was performed on 46 candidates for bariatric surgery with a mean body mass index of 46.5 kg/m^2^. Esophagogastric junction outflow obstruction was diagnosed in 16 (34.8%) patients, and ineffective esophageal motility was diagnosed in 8 (17.4%) patients. None of the subjects reported symptoms during the preoperative period. Out of the 46 individuals initially included, 44 underwent bariatric surgery, 23 (52.3%) underwent Roux-en-Y gastric bypass, and 21 (47.7%) underwent sleeve gastrectomy. One patient with esophagogastric junction outflow obstruction reported dysphagia after Roux-en-Y bypass, but symptoms spontaneously resolved during the 90-day follow-up period.

**CONCLUSIONS:**

Although patients with severe obesity have a high prevalence of esophageal motility disorders, no clinical repercussions were observed after bariatric surgery during the study period.

## RESUMO

**RACIONAL:**

Evidências recentes mostram que a obesidade está associada a doença do refluxo gastroesofágico (DRGE) e a dismotilidade esofágica, embora os sintomas nem sempre estejam presentes.

**OBJETIVOS:**

Estudo prospectivo baseado nos achados de manometria de alta resolução em pacientes obesos e sua correlação com disfagia após a cirurgia bariátrica.

**MÉTODOS:**

A avaliação manométrica foi realizada nos candidatos a cirurgia bariátrica, de 2022 a 2024. O exame foi conduzido de acordo com o protocolo da quarta versão da Classificação de Chicago, incluindo diferentes posições e manobras provocativas para confirmar os diagnósticos de dismotilidade. Os pacientes foram acompanhados por 90 dias após a cirurgia para verificar a ocorrência de disfagia ou dificuldade de adaptação à dieta.

**RESULTADOS:**

A manometria de alta resolução foi realizada em 46 pacientes com índice de massa corporal médio de 46,5 kg/m2. O distúrbio de obstrução ao fluxo de saída da junção esofagogástrica foi diagnosticado em 16 (34,8%) pacientes, enquanto motilidade esofágica ineficaz foi diagnosticada em 8 (17,4%) pacientes. Nenhum paciente relatou sintomas no período pré-operatório. Dos 46 indivíduos incluídos incialmente, 44 realizaram cirurgia bariátrica, sendo que 23 (52,3%) foram submetidos ao *by-pass* gástrico em Y-de-Roux e 21 (47,7%) à gastrectomia vertical. Um paciente diagnosticado com obstrução ao fluxo de saída da junção esofagogástrica relatou disfagia após *by-pass* gástrico, porém apresentou resolução espontânea dos sintomas durante o período de seguimento pós-operatório.

**CONCLUSÕES:**

Embora os pacientes com obesidade grave apresentem prevalência elevada de distúrbios de motilidade esofágica, não foram observadas repercussões clínicas após a cirurgia bariátrica durante o período da pesquisa.

## INTRODUCTION

Adult obesity has more than doubled since 1990 and is a major health problem worldwide. The consequences of obesity are well documented and understood. Most deaths in patients with a higher-than-optimal body mass index (BMI) are caused by cardiovascular diseases, diabetes, and cancer^13^. There is also evidence that obesity is associated with less severe gastroesophageal diseases, including gastroesophageal reflux disease (GERD) and esophageal dysmotility, even though symptoms are not always present^4,8^.

Bariatric surgery is currently the most effective and durable treatment for obesity. Roux-en-Y gastric bypass and sleeve gastrectomy are the two main techniques of choice^1,6^. Although routine evaluation of motility disorders before bariatric surgery is still a subject of debate^9,17^, derangement in the anatomy of the stomach and the esophagogastric junction (EGJ) may cause consequences on the physiology of the esophagus. New onset of GERD or worsening of preexisting symptoms, for example, can occur after sleeve gastrectomy^20^. Recently, esophageal dysmotility and postoperative dysphagia were also suggested to be common long-term complications after bariatric surgery, including manometric findings of an achalasia-like pattern^12^. However, the correlation between high-resolution impedance manometry (HRIM) and clinically significant symptoms is not yet completely established in this population^11,14,19^.

In recent years, the popularization of HRIM amplified the comprehension of esophageal motility. More recently, the Chicago Classification Scheme for esophageal motility disorders on HRIM has been updated to its fourth version (CCv4.0), incorporating different test positions and provocative tests^7,18^. These changes attempt to minimize ambiguity and provide more standardized and rigorous criteria for determining patterns of peristalsis disorders and obstruction at the EGJ, especially when applied to individuals with obesity^15^. This study aims to prospectively assess the characteristics of esophageal motility disorders in patients planning to undergo bariatric surgery according to the CCv4.0 protocol and correlate them with postoperative dysphagia.

## METHODS

### Study protocol

A prospective study was conducted at our tertiary academic center from May 2022 to February 2024. Every candidate for bariatric and metabolic surgery who agreed with the consent protocol was included in the study and underwent a preoperative HRIM. These patients were older than 18 years and had either a BMI of 40 or 35 with obesity-related comorbidities. Patients who had a prior history of upper gastrointestinal surgery or scleroderma were excluded. The study obtained approval from the institutional Research Ethics Committee (number 57139622.9.0000.532), and a waiver of informed consent was obtained from all patients.

All surgery candidates routinely underwent preoperative esophagogastroduodenoscopy with gastric biopsies for the exclusion of *Helicobacter pylori*. The HRIM was scheduled 1 week before the surgery date. Because we lack a validated dysphagia questionnaire in Portuguese, we investigated symptoms by formulating questions based on the Brief Esophageal Dysphagia Questionnaire (BEDQ). In the exam interview, patients were asked about the presence of dysphagia due to drinking liquids, eating soft or solid food, as well as choking or pain while swallowing.

Patients were then submitted to either Roux-en-Y bypass or sleeve gastrectomy according to the evaluation of the bariatric surgery team. All procedures were performed by the same specialized surgeons (MRMT, ENT, and LSD). In the postoperative period, patients had the first follow-up appointments at 14, 30, and 90 days with the multidisciplinary team. At these visits, patients were also investigated for dysphagia or symptoms concerning diet adaptation.

### High-resolution impedance manometry

HRIM was performed and analyzed according to the CCv4.0 protocol by a single gastroenterologist (ABL). Before the examinations, the participants fasted for 8 h. HRIM was performed using a solid-state catheter with 32 circumferential pressure transducers at 1-cm intervals and dual impedance sensors (Sandhill Scientific, Highlands Ranch, CO, USA). Esophageal body motility was assessed with 10 liquid swallows of 5 mL at 30-s intervals in the supine position. The multiple rapid swallow (MRS) test was performed two times, which involved drinking 2 mL of water for five successive swallows, separated by 2–3 s intervals in the supine position. Another five liquid swallows were recorded in the upright, seated position. The rapid drinking challenge (RDC) was the last test performed, which involved drinking 200 mL of water as fast as possible in the upright position.

The captured HRIM parameters included EGJ baseline pressure, integrated relaxation pressure (IRP), distal contractile integral (DCI), distal latency, EGJ morphology, and impedance values. BioView (Sandhill Scientific) analysis software was used to interpret the data, and the same gastroenterologist reviewed all HRIM tracings. Motility disorders were defined according to CCv4.0. The diagnosis of esophagogastric junction outflow obstruction (EGJOO) was defined as an elevated IRP both in supine (15 mmHg) and upright (12 mmHg) positions with the evidence of peristalsis. Contractile patterns were based on the 10 supine swallows and considered hypercontractile if 20% swallows with distal contractile integral >8,000 mmHg/cm/s, ineffective if >70% ineffective or 50% failed swallows, or normal peristalsis if neither of the former. The MRS and RDC provocative tests were used in an attempt to improve the specificity of manometric diagnosis. The absence of a contraction reserve, defined by a DCI following MRS greater than a single swallow mean DCI, was considered supportive of ineffective esophageal motility (IEM). A bolus transit of <50% on impedance was considered a poor result and supportive of IEM. An IRP >12 mmHg in both MRS and RDC was considered supportive of EGJOO.

### Statistical analysis

Descriptive statistics for continuous measures were presented as means (standard deviation [SD]) or medians (interquartile range [IQR]), respectively, when normal or non-normal distribution was observed. The one-way ANOVA and Kruskal-Wallis tests were used to analyze quantitative variables according to the distribution of data. Both tests were applied for group comparison of categorical variables. Associations between supine IRP and weight and BMI were evaluated using Spearman’s correlation. Statistical significance was denoted when p<0.05. All statistical analyses were performed using SPPS Statistics v18.0 (SPSS Inc., 2009, Chicago, USA).

## RESULTS

A total of 47 consecutive candidates for bariatric surgery were selected to undergo preoperative HRM. One patient was unable to tolerate the exam and was excluded from the analysis. Baseline characteristics are outlined in Table 1. The mean age was 50.7 (±12) years, and 87% of the patients were females. The median BMI was 46.5 (43.1–54) kg/m^2^ and the highest was 79.7 kg/m^2^ (206.5 kg). Half of the patients had type 2 diabetes, and 76% had hypertension. All patients underwent preoperative endoscopy, and 6 (13%) showed signs of gastroesophageal reflux disease (esophagitis Los Angeles grades B, C, or D).

**Table 1:** Baseline characteristics.

Characteristic	n=46
Age, years – mean (SD)	50.7 (12)
Female – n (%)	40 (87)
BMI, kg/m^2^ – median (IQR)	46.5 (43.1–54)
Weight, kg – median (IQR)	122.5 (106.4–149)
Smoking – n (%)	5 (10.9)
Hypertension – n (%)	35 (76.1)
Type 2 diabetes – n (%)	23 (50)
Opioid use – n (%)	4 (8.7)
Proton pump inhibitors use – n (%)	10 (21.7)
Endoscopy findings – n (%)	
LA grades B, C, and D	6 (13)
LA grade A	3 (6.5)
Surgery – n (%)	44 (95.6)
Roux-en-Y bypass	23 (50)
Sleeve gastrectomy	21 (45.6)

SD: standard deviation; BMI: body mass index; kg: kilogram; IQR: interquartile range; LA: Los Angeles Classification.

**Figure 1:**
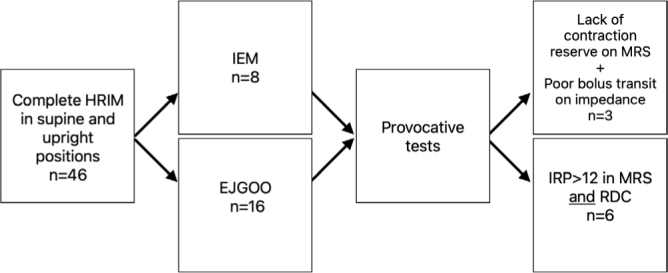
High-resolution manometry protocol performed in bariatric surgery candidates based on CCv4.0.

HRM: high-resolution manometry; IEM: ineffective esophageal motility; EGJOO: esophagogastric junction outflow obstruction; IRP: integrated relaxation pressure; MRS: multiple rapid swallow; RDC: rapid drink challenge.

Out of the 46 individuals who completed the preoperative HRIM, 24 (52.2%) had an elevated IRP in the supine position. Considering results in both supine and upright positions, 16 (34.8%) had a manometric diagnosis of EGJOO and 8 (17.4%) had IEM according to CCv4.0. Within the EGJOO group, three patients exhibited a peristaltic pattern of IEM, and one had a hypercontractile esophagus. After a series of provocative tests, there was supportive evidence of EGJOO in 6 (13%) patients and IEM in 3 (6.5%) patients (Figure 1). Patients were categorized into subgroups according to the manometric diagnosis, and their characteristics were compared. There were no statistical differences in weight, BMI, or risk factors between the groups (Table 2). Spearman’s analysis showed that there were no correlations between supine IRP and weight or BMI (Table 3).

**Table 2:** Subgroup characteristics comparison.

Characteristics	Nº HRIM alterations (n=22)	EGJOO (n=16)	IEM (n=8)	p-value
Age, years – mean (SD)	50.6 (12.6)	53.9 (11.2)	44.7 (10.7)	0.212
Female – n (%)	19 (86.4)	13 (81.3)	8 (100)	0.555
BMI, kg/m^2^ – median (IQR)	46.5 (43.3–51.9)	47.7 (43.1–58)	44.4 (39.3–53.2)	0.567
Weight, kg – median (IQR)	119.8 (109.4–144)	134.1 (107.7–163.4)	113.8 (99.1–137.7)	0.397
Smoking – n (%)	1 (4.5)	4 (25)	0	0.104
Hypertension – n (%)	15 (68.2)	15 (93.8)	5 (62.5)	0.104
Type 2 diabetes – n (%)	12 (54.5)	8 (50)	3 (37.5)	0.792
Opioid use – n (%)	0	3 (18.8)	1 (12.5)	0.116
Proton pump inhibitors use – n (%)	6 (27.3)	2 (12.5)	2 (25)	0.621
Endoscopy findings – n (%)				
LA grades B, C, and D	4 (18.2)	1 (6.3)	1 (12.5)	0.741
LA grade A	3 (13.6)	0	0	0.212

SD: standard deviation; BMI: body mass index; kg: kilogram; IQR: interquartile range; LA: Los Angeles Classification; HRIM: high-resolution impedance manometry; EGJOO: esophagogastric junction outflow obstruction; IEM: ineffective esophageal motility.

**Table 3:** Spearman’s correlation of supine integrated relaxation pressure with weight and body mass index.

	Correlation coefficient	p-value
Weight	0.103	0.497
BMI	-0.031	0.835

BMI: body mass index.

**Figure 2:**
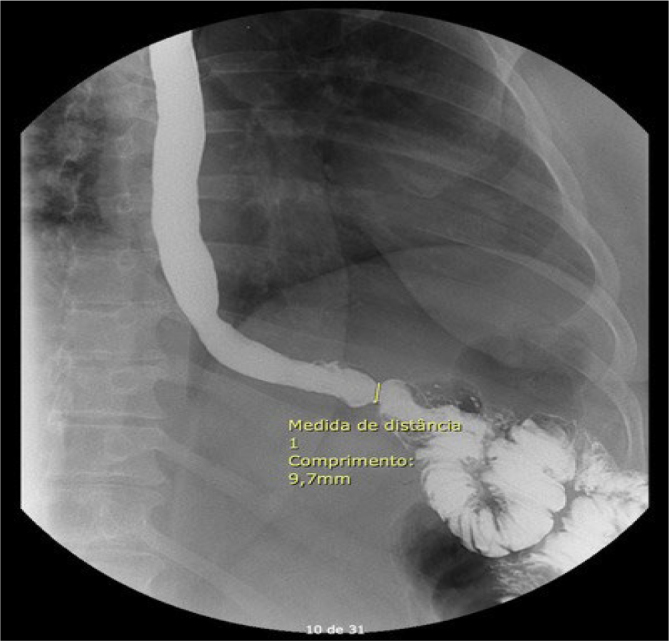
Postoperative barium esophagogram of the gastric bypass patient with preoperative findings of esophagogastric junction outflow obstruction that reported dysphagia when started introducing solid foods. The esophagogastric junction’s width is 9.7 mm. There was no anastomotic stricture.

During the study, one patient abandoned, and another postponed the surgery. None of the 44 individuals submitted to bariatric surgery reported dysphagia preoperatively. Roux-en-Y gastric bypass was performed in 23 patients and sleeve gastrectomy in 21 patients. In the first postoperative month, only one bypass patient with manometric findings of EGJOO confirmed in the provocative tests, and no disorder of peristalsis reported trouble when starting the solid diet. Subsequent investigation showed a barium esophagogram with a narrow passage of 9.7 mm in the lower esophageal sphincter (Figure 2). Upper gastrointestinal endoscopy revealed no alterations or anastomotic stricture. The patient was instructed to stay on a soft diet for a longer period than the protocol and gradually introduce solids. Within 3 months, the patient reported improvement in dysphagia and fully adapted to the solid diet.

## DISCUSSION

In this study, we prospectively assessed esophageal motility disorders in patients with obesity and the occurrence of dysphagia after bariatric surgery. Considering the CCv4.0, we found that manometric alterations occurred in 52.2% of bariatric surgery candidates. EGJOO was the most common diagnosis, presented in 34.8% of the patients. However, these findings were not clinically relevant once only one patient reported transitory dysphagia in the postoperative period.

Previous studies have already demonstrated high rates of manometric abnormalities in patients with obesity^4,8^. To the best of our knowledge, this is the first prospective study to utilize HRIM and the last version of the Chicago Classification as a routine workup before bariatric surgery. One of the CCv4.0 key updates was the incorporation of supine and upright positions for the diagnosis of EGJOO. Su et al. demonstrated that an artificial elevation in IRP is common in patients with obesity due to increased abdominal pressure and that position changing is important because of the abdominal fat redistribution^15^. More recently, Flanagan et al., however, hypothesized that the IRP may sometimes be falsely low in patients with obesity due to the high gastric baseline pressure, which has an inverse relationship with IRP. That was demonstrated by the discrepancy between functional lumen imaging probe (FLIP) and HRIM results in their findings^5^. In our study, we could not find a correlation between elevated supine IRP and weight or BMI, probably due to the sample size. Additionally, the patient with the highest BMI in our cohort had a completely normal HRIM. Even so, we found that 35% of patients with elevated IRP in the supine position had normalized IRP in the upright position, suggesting that position changing is important for preventing the overdiagnosis of EGJOO in individuals with obesity.

It is not completely understood whether the diagnosis of EGJOO is a clinically relevant problem or not. Most of the patients who present with this manometric diagnosis are asymptomatic, as reported previously^2,3^. The new CCv4.0 tries to resolve this dilemma by demanding the presence of symptoms and confirming obstruction by timed barium esophagogram (TBE) or FLIP to characterize a clinically relevant diagnosis. In our study, none of the patients with manometric findings of EGJOO reported symptoms of swallowing obstruction preoperatively. One patient with EGJOO and an apparent obstruction in the barium esophagogram reported dysphagia for a solid diet after gastric bypass, but the symptoms were mild and transitory. Time likely plays a crucial role in the development of esophageal motility alterations. In the study of Miller et al., postoperative dysphagia emerged as a long-term complication of bariatric surgery, with evidence indicating the development of esophageal dysmotility and achalasia-like patterns in HRIM^12^. Thus, longer follow-up periods can be necessary to determine whether patients with obesity and HRIM alterations will develop significant esophageal changes after bariatric surgery.

Another major finding was that 37.5% of the patients with EGJOO or IEM were excluded from the diagnosis when considering the results of MRS, RDC, and bolus transit on impedance. Krause et al. found that these provocative tests are adjunctive HRIM maneuvers that appear to help identify clinically significant EGJOO^10^. They are also incorporated as supportive tests in CCv4.0, but they are not strictly necessary for the diagnosis of motility disorders. Our intention was to verify if these maneuvers could increase the specificity of manometric diagnosis in patients with obesity. Notably, the only symptomatic patient in our study had EGJOO confirmed with the RDC, supporting the value of provocative maneuvers during the HRIM examination.

We designed a prospective study and utilized the latest HRIM classification protocol available; however, our study has some limitations. First, our statistical analysis was likely affected by the sample size. Additionally, symptoms were investigated based on the BEDQ, which is a reliable assessment tool for esophageal dysphagia^16^. However, we lacked access to this validated questionnaire in Portuguese, so we could not compare scores before and after surgery. Finally, while a longer follow-up period may be necessary to fully observe the development of esophageal dysfunction, our study lays a strong foundation for future research. It is important to acknowledge that manometric evaluation, although invasive and complex, has been effectively applied in this cohort, providing valuable insights into the esophageal motility of obese subjects. Ideally, future studies should also assess HRIM results before and after bariatric surgery in the same cohort.

## CONCLUSION

Patients with severe obesity present a high prevalence of HRIM abnormalities. EGJOO is a common finding, probably due to higher intra-abdominal pressure or artifacts caused by abdominal fat distribution. Position changing and provocative maneuvers can help increase the specificity of manometric diagnosis. The absence of postoperative symptoms suggests that these alterations may not be clinically relevant in the context of bariatric surgery. Larger studies with comparative HRIM results and a longer follow-up period are necessary to confirm changes in esophageal anatomy and function after bariatric procedures.
